# Gut microbial metabolites reveal diet-dependent metabolic changes induced by nicotine administration

**DOI:** 10.1038/s41598-024-51528-3

**Published:** 2024-01-11

**Authors:** Ryuji Ohue-Kitano, Yukika Banno, Yuki Masujima, Ikuo Kimura

**Affiliations:** 1 Laboratory of Molecular Neurobiology, Division of Systemic Life Science, Graduate School of Biostudies, Kyoto University, Sakyo-ku, Kyoto, 606-8501 Japan; 2 Laboratory of Molecular Endocrinology, Division of Medicinal Frontier Sciences, Graduate School of Pharmaceutical Sciences, Kyoto University, Sakyo-ku, Kyoto, 606-8501 Japan; 3 Center for Living Systems Information Science (CeLiSIS), Kyoto University, Sakyo-ku, Kyoto, 606-8501 Japan; 4 Department of Applied Biological Science, Graduate School of Agriculture, Tokyo University of Agriculture and Technology, Fuchu, Tokyo 183-8509 Japan

**Keywords:** Homeostasis, Metabolic diseases, Microbiology, Endocrinology, Nutrition

## Abstract

The gut microbiota has emerged as an important factor that potentially influences various physiological functions and pathophysiological processes such as obesity and type 2 diabetes mellitus. Accumulating evidence from human and animal studies suggests that gut microbial metabolites play a critical role as integral molecules in host–microbe interactions. Notably, several dietary environment-dependent fatty acid metabolites have been recognized as potent modulators of host metabolic homeostasis. More recently, nicotine, the primary active molecule in tobacco, has been shown to potentially affect host metabolism through alterations in the gut microbiota and its metabolites. However, the mechanisms underlying the interplay between host nutritional status, diet-derived microbial metabolites, and metabolic homeostasis during nicotine exposure remain unclear. Our findings revealed that nicotine administration had potential effects on weight regulation and metabolic phenotype, independent of reduced caloric intake. Moreover, nicotine-induced body weight suppression is associated with specific changes in gut microbial composition, including *Lactobacillus* spp., and KetoB, a nicotine-sensitive gut microbiota metabolite, which could be linked to changes in host body weight, suggesting its potential role in modulating host metabolism. Our findings highlight the remarkable impact of the interplay between nutritional control and the gut environment on host metabolism during smoking and smoking cessation.

## Introduction

The gut microbiota plays a crucial role in several physiological functions, including metabolic homeostasis and the development of metabolic disorders, such as obesity and type 2 diabetes mellitus^1^. The gut microbiota can produce a wide range of bioactive metabolites that serve as sensitive indicators of microbial function and signal messengers for the host microbiome by entering the bloodstream and tissues. In addition, gut microbiota is susceptible to daily environmental factors, and diet is a potent modulator of microbiota composition and function^2^. Diet-derived metabolites produced by gut bacteria can substantially affect host metabolism. Short-chain fatty acids (SCFA) derived from the fermentation of indigestible polysaccharides by gut microbes are prominent dietary metabolites associated with improved insulin sensitivity and weight gain resistance^3^. The gut microbiota can promote the saturation of dietary polyunsaturated fatty acids, which synthesize diverse fatty acid variants such as conjugated-, hydroxy-, and oxy-fatty acids^4^. These metabolites exert various physiological effects and functions, including conferring host resistance to high-fat diet (HFD)-induced obesity^5^ and promoting energy expenditure by activating the sympathetic nervous system^6^. Thus, fatty acid metabolites dependent on the dietary environment of the host are considered effective molecules for enhancing host metabolic functions and establishing mutually beneficial symbiotic relationships.

Cigarette smoking is currently a primary contributor to preventable fatalities worldwide. Approximately 50% of smokers are susceptible to severe smoking-related ailments, such as chronic obstructive pulmonary disease, cardiovascular issues, and various cancers^7–9^. Exposure to secondhand smoke has been shown to increase the risk of pathogen-related infections and exacerbate conditions like asthma^10^. Additionally, smoking has a dual impact on inflammatory bowel disease, aggravating Crohn’s disease while ameliorating the symptoms associated with ulcerative colitis^11^. The harmful constituents of cigarette smoke are widely regarded as major factors in serious diseases, prompting an extensive investigation of the underlying pathological mechanisms^12^. However, these mechanisms remain unclear. Nicotine, the primary active molecule in tobacco, is typically inhaled into the lungs where it is rapidly absorbed by the pulmonary alveoli. It can also be absorbed through the skin and gastrointestinal tract. Nicotine can have multiple beneficial and detrimental effects on host metabolism, including potentiation of metabolic rate, regulation of energy intake by modulating appetite, suppression of lipid storage in adipose tissue by influencing lipolysis, enhancement of energy expenditure by increasing sympathetic drive and thermogenesis, and development of cardiovascular diseases and hepatic steatosis triggered by HFD^13^. These effects are primarily attributed to the modulation of hypothalamic neuropeptide systems such as proopiomelanocortin and energy sensors such as AMP-activated protein kinase, which function in the central nervous system^14,15^. In addition, nicotine has been shown to directly influence peripheral metabolic tissues, such as brown adipose tissue, white adipose tissue (WAT), the liver, and the pancreas^13^. Thus, nicotine has been suggested to play a significant role in metabolic homeostasis through numerous biological processes.

Cigarette smoking and cessation affect the gut environment and may lead to alterations in the commensal microbial community, including the gut microbiota composition and metabolite profiles^16^. In addition, extensive epidemiological studies have provided compelling evidence of a robust association between cigarette smoking, smoking cessation, and body weight fluctuation^17^. Interestingly, it has been reported that weight gain following smoking cessation may be associated with a transition to a gut microbiota profile similar to the recently identified "obese microbiota" observed in both human and animal studies^18^. Furthermore, Fluhr et al.^19^ demonstrated that smoking and cessation disrupt the microbial balance, followed by several gut microbial metabolites such as dimethylglycine and *N*-acetylglycine, which are correlated with nicotine-induced body weight fluctuations. In addition, indole, a gut bacterial metabolite derived from tryptophan, increased following nicotine administration, and attenuated dextran sulfate sodium-induced colitis^20^. According to Chen et al., nicotine accumulates within the gastrointestinal tract during smoking, leading to the development of nonalcoholic fatty liver disease. Additionally, they determined that the gut bacterium *Bacteroides xylanisolvens* could degrade nicotine to 4-hydroxy-1-(3-pyridyl)-1-butanone, potentially relieving hepatic diseases^21^. Thus, nicotine exposure has been suggested to regulate host metabolism and dysfunction through changes in the gut microbiota and its metabolites. However, the mechanisms underlying the interplay between the gut nutritional environment, subsequent diet-derived microbial metabolites, and host metabolic homeostasis during nicotine exposure remain unclear.

Therefore, this study aimed to investigate the effect of nicotine on metabolic regulatory mechanisms by manipulating the intestinal environment, including the nutritional milieu and gut microbial composition, and to elucidate the underlying molecular mechanism responsible for these observed effects using different mouse models. Furthermore, the effect on metabolic phenotypes has been shown to vary depending on the acute or chronic administration of nicotine. Thus, a global meta-analysis of the gut microbiota and its metabolites across nicotine exposure and delivery methods was conducted to elucidate the detailed mechanisms through which nicotine exposure-induced gut microbiota changes affect host metabolism. In this study, we demonstrated that intraperitoneal nicotine administration affects weight control and biochemical characteristics independently of the effects of reduced caloric intake; these effects were associated with the modulation of specific gut bacteria, corresponding to the involvement of microbiota metabolites in a diet-dependent manner.

## Results

### Remarkable alterations of metabolic profile induced by intraperitoneal nicotine administration during HFD consumption

**Figure 1:**
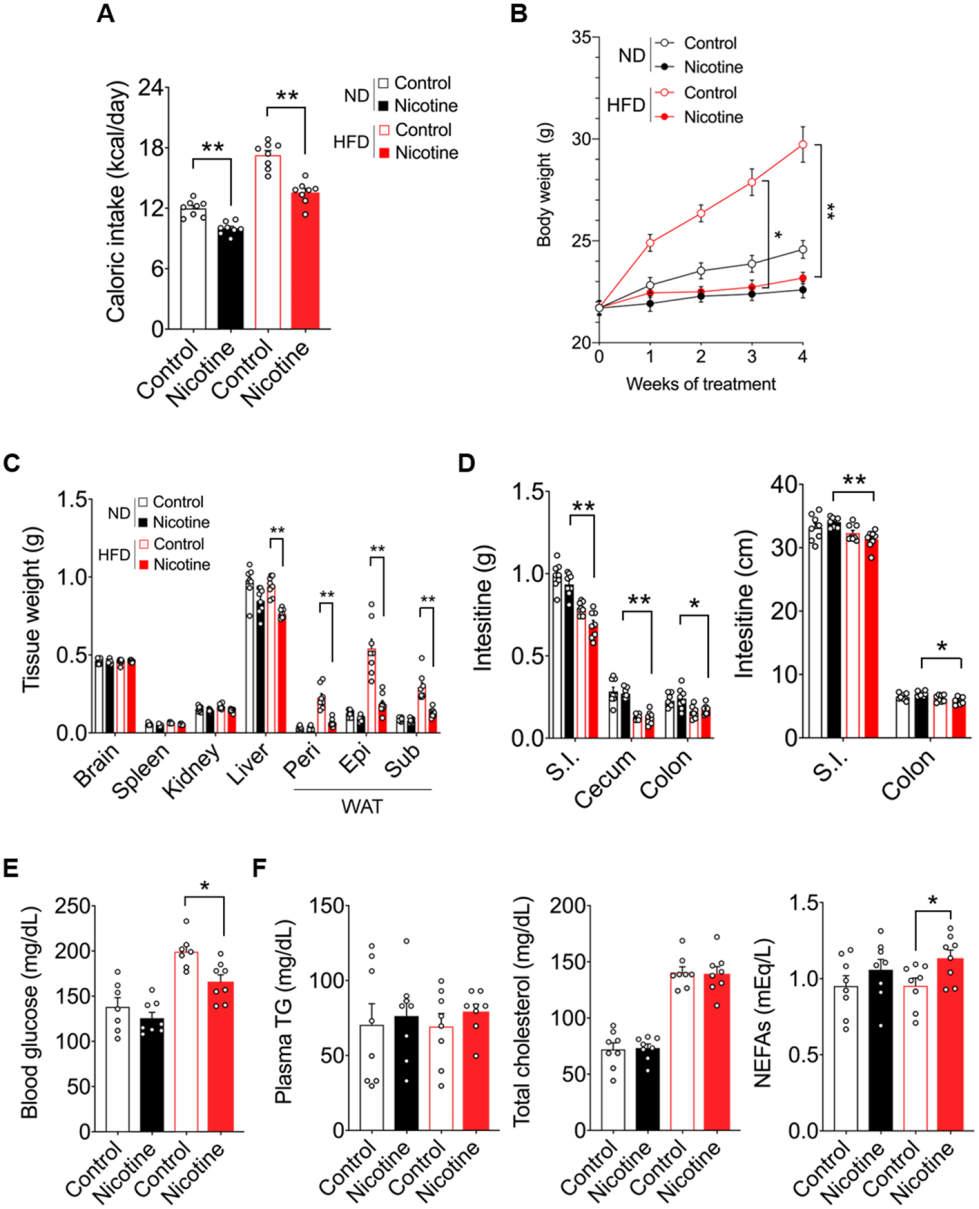
Diet-dependent changes in metabolic parameters during nicotine treatment. C57BL/6J wild-type male mice were fed ND or HFD for four weeks under nicotine exposure condition. (**A**) Daily caloric intake (n = 8 per group), (**B**) bodyweight gain (n = 8 per group), (**C**) mass of the brain, spleen, kidney, liver, and WAT (n = 8 per group), and (**D**) mass and size of the intestinal tract (n = 8 per group), (**E**) blood glucose levels (n = 8 per group), (**F**) plasma levels of TG, total cholesterol, and NEFAs (n = 8 per group). All data are presented as the mean ± standard error of the mean. **P < 0.01; *P < 0.05 (one-way ANOVA followed by post hoc Tukey’s test: A, C, E, F; Kruskal–Wallis test followed by post hoc Dunn’s test: D; Two-way ANOVA followed by post hoc Bonferroni test: B). ND, normal diet; HFD, high-fat diet; WAT, white adipose tissue; Peri, perirenal white adipose tissue; Epi, epididymal white adipose tissue; Sub, subcutaneous white adipose tissue; SI, small intestine; TG, triglyceride; NEFA, non-esterified fatty acids.

To investigate the effects of nicotine on the metabolic parameters associated with the increase in blood nicotine concentration, excluding the direct effects of nicotine on the gut; we first administered nicotine (1.5 mg/kg bodyweight) intraperitoneally to 7-week-old C57BL/6J wild-type mice which were fed a normal diet (ND) or HFD for four weeks. Notably, administering a daily dose of 1.5 mg/kg bodyweight to mice results in a serum nicotine concentration that reflects clinically relevant levels observed in habitual cigarette smokers or users of nicotine-containing chewing gum^15^. Intraperitoneal nicotine administration resulted in a significant decrease in daily caloric intake in both ND- [from control group (11.99 kcal/day) to nicotine group (10.00 kcal/day)] and HFD mice [from control group (17.28 kcal/day) to nicotine group (13.56 kcal/day)] (Fig. 1A). In accordance with the caloric intake findings, body weight gain was substantially decreased in nicotine-treated mice during growth (Fig. 1B). Interestingly, the extent of weight loss induced by nicotine administration was greater in the HFD group than in the ND group. The masses of the liver and WAT were also significantly lower in nicotine-treated mice than in control mice under HFD-fed conditions; however, no notable difference was observed in the weights of the brain, spleen, and kidney (Fig. 1C). Contrarily, in the ND-fed mice, the tissue weights of the liver and WAT showed hardly a moderate decrease between the nicotine-treated and control mice. Interestingly, in the morphological feature of the intestinal tract, the weight and size of the small intestine and colon were significantly lower in the HFD group compared with the ND group after nicotine administration (Fig. 1D). In biochemical analysis, a significant reduction in blood glucose was observed in HFD-fed mice alone after nicotine administration (Fig. 1E). In HFD-fed mice, intraperitoneal administration of nicotine resulted in a significant increase in plasma non-esterified fatty acid (NEFA) levels but not in regard to the total cholesterol or triglyceride (TG) levels (Fig. 1F). In contrast, no notable changes in any of the lipid metabolism parameters were observed in ND-fed mice. Therefore, the effect of nicotine administration on metabolic phenotype may be diet dependent. Although the marked decrease in caloric intake during HFD feeding conditions may potentially contribute to the alteration of body weight and metabolic profiles in mice, further investigation is warranted to understand why these distinctive phenotypes occur during HFD consumption alone and how they affect metabolic homeostasis.

### The effects of nicotine administration on metabolic parameters during the controlled feeding regimen

**Figure 2:**
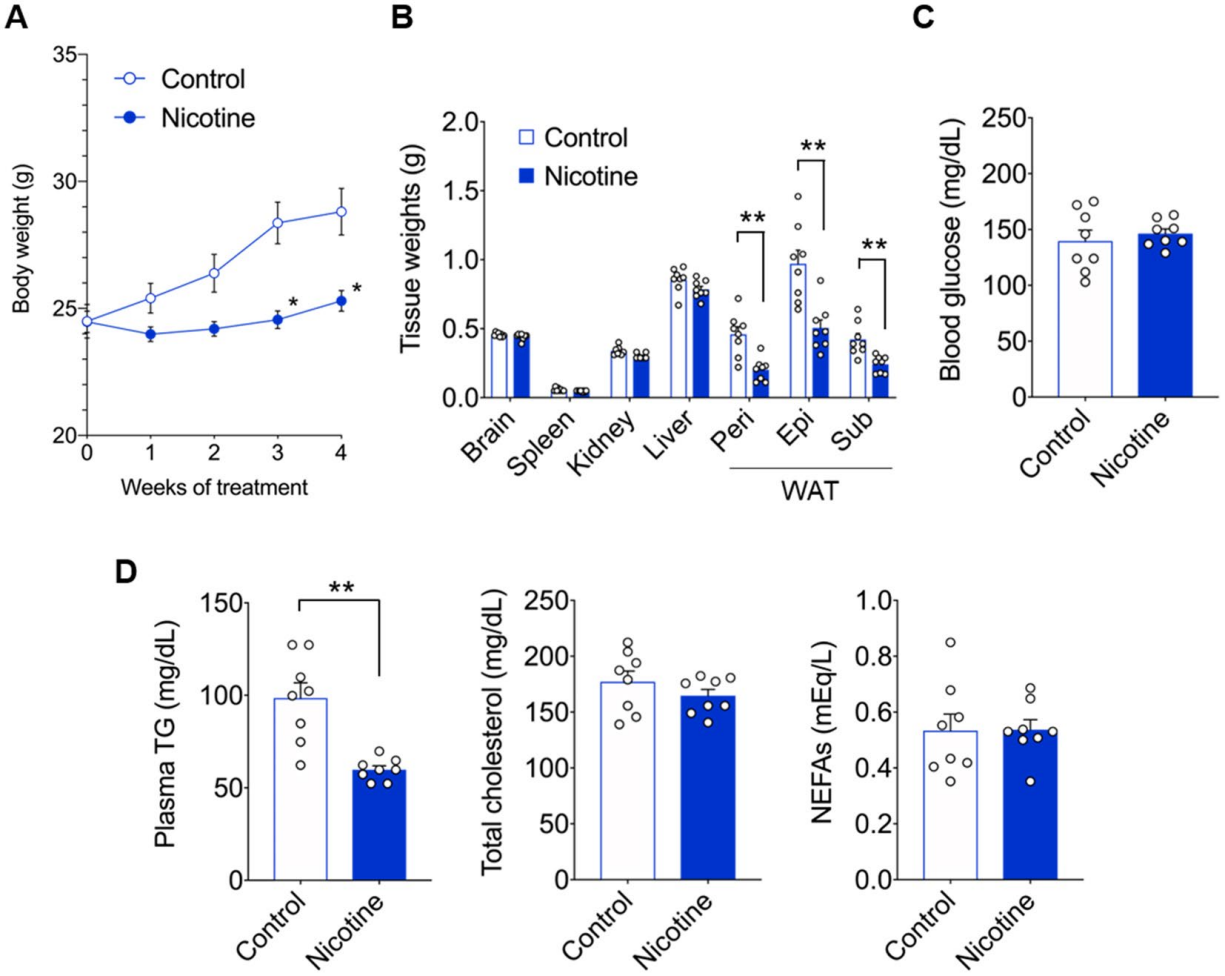
Changes in metabolic parameters associated with nicotine administration under pair-feeding conditions. C57BL/6J wild-type male mice were fed HFD for four weeks under nicotine exposure condition. Control mice were received saline. (**A**) Bodyweight gain (n = 8 per group), (**B**) mass of the brain, spleen, kidney, liver, and WAT (n = 8 per group), (**C**) blood glucose levels (n = 8 per group), (**D**) plasma TG, total cholesterol, and NEFAs (n = 8 per group). All data are presented as the mean ± standard error of the mean. **P < 0.01; *P < 0.05 (Two-way ANOVA followed by post hoc Bonferroni test: A; Mann–Whitney U test: B–D). HFD, high-fat diet; WAT, white adipose tissue; Peri, perirenal white adipose tissue; Epi, epididymal white adipose tissue; Sub, subcutaneous white adipose tissue; TG, triglyceride; NEFA, non-esterified fatty acids.

We evaluated the effects of HFD intake and nicotine exposure on metabolic phenotypes in a paired-feeding model with controlled and standardized caloric intake. As shown in Fig. 2A, nicotine-treated mice showed marked suppression of body weight gain compared to control mice, despite the standardization of caloric intake under HFD feeding. In addition, although the weight of WAT was significantly lesser in nicotine-treated mice than in control mice, there was no notable difference in liver weight between the control and nicotine-treated groups, in contrast to the ad libitum feeding conditions (Fig. 2B). In contrast, no notable differences were observed between the control and nicotine-treated mice in blood glucose or plasma levels of total cholesterol and NEFAs, except for plasma TG (Fig. 2C, D). Therefore, in the pair-feeding model with HFD, nicotine administration resulted in alterations in blood biochemical parameters but consistently suppressed weight gain, indicating the involvement of additional factors beyond the regulation of caloric intake in the metabolic phenotype induced by intraperitoneal nicotine exposure.

### Nicotine administration leads to changes in gut microbiota specific to HFD feeding conditions

**Figure 3:**
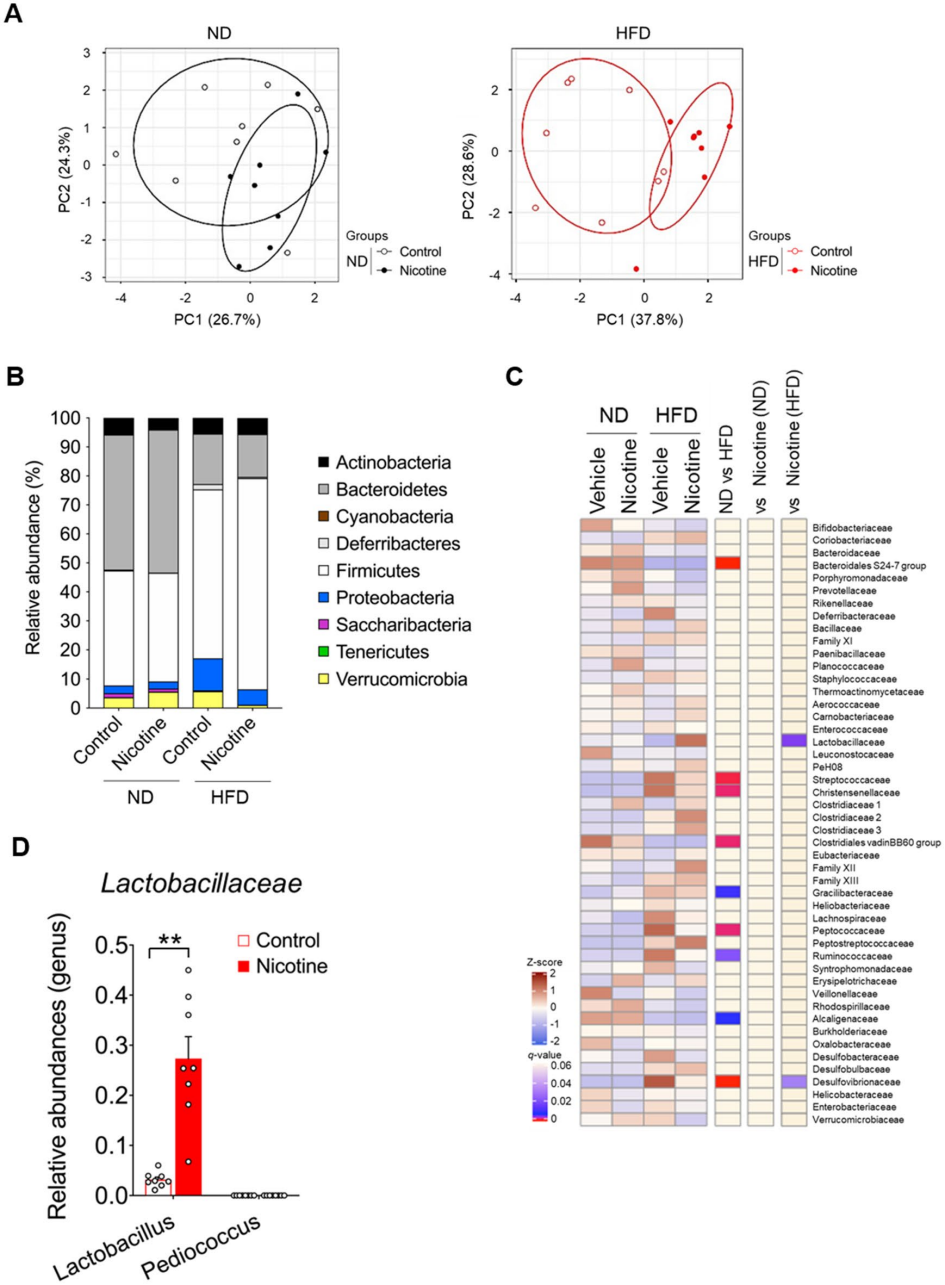
Altered gut microbiota composition in mice fed HFD versus ND following nicotine administration. (**A**) Principal coordinate analysis of the fecal microbiota in the four-week period of intraperitoneal nicotine-treated mice based on unweighted Unifrac distances between diet groups (n = 7–8). (**B**) Relative abundance of the phylum level (n = 7–8). (**C**) Heatmap of relative abundance of major taxonomic groups at family level (n = 7–8). FDR, *q* < 0.1. *q*-values were estimated using the Benjamini–Hochberg procedure. (**D**) The abundance of the genus *Lactobacillaceae*. All data are presented as the mean ± standard error of mean. **P < 0.01; *P < 0.05 (Permutational multivariate analysis of variance tests: A; Student’s t-test: D). ND, normal diet; HFD, high-fat diet.

Extensive evidence suggests that nicotine affects gut health and may lead to changes in the commensal bacteria, which may have effects on the overall health^16,22,23^. Then, we examined the changes in the gut microbial composition following nicotine administration in mice. After examining ND- and HFD-fed mice for four weeks, we noted that intraperitoneal nicotine administration altered the gut microbial composition, as indicated by principal coordinate analysis (PCA) and clustering analysis based on taxonomic datasets. Nicotine exposure significantly shifted the structure of the gut bacterial community in HFD-fed mice (*P* = 0.013); however, the shift induced by nicotine was moderate in ND-fed mice (*P* = 0.638; Fig. 3A). Taxonomic analysis of the fecal microbiota revealed an increased abundance of Firmicutes and reduced populations of Deferribacteres, Proteobacteria, and Verrucomicrobia in nicotine-treated HFD-fed mice (Fig. 3B). The relative abundance of Firmicutes was significantly higher in the nicotine group than in the control group (*P* = 0.005), whereas the relative abundances of Deferribacteres, Proteobacteria, and Verrucomicrobia tended to decrease (*P* = 0.077, *P* = 0.001, and *P* = 0.075, respectively; Fig. 3B). Intraperitoneal nicotine administration did not affect the relative abundance of any other phyla. Furthermore, the hierarchical clustering of individual families in nicotine-treated HFD-fed mice exhibited a marked increase in *Lactobacillaceae*, along with a substantial decrease in *Desulfovibrionaceae* (Fig. 3C). In particular, the abundance of *Lactobacillus* significantly increased in nicotine-treated mice compared to that in control mice in the HFD-fed group (Fig. 3D). To determine the potential impact of direct oral nicotine delivery models on gut microbiota dynamics, we chronically administered nicotine in water ad libitum to the intestines of the HFD-fed mice (approximately 1348.79 ± 95.53 µg/day of nicotine). Despite the marked reduction in caloric intake and the observed inhibition of weight gain and mass of WAT following oral nicotine administration (Fig. S1A–C) similar to what was observed with intraperitoneal administration of nicotine, no alterations in blood glucose levels were observed (Fig. S1D). In accordance with the disparities in metabolic phenotypes, discernible differences in the gut microbiota composition at the phylum level were noted between the two nicotine administration methods. Oral administration of nicotine also led to notable changes in the composition of the gut microbiota (*P* = 0.027; Fig. S2A). In contrast to intraperitoneal nicotine administration, oral nicotine administration resulted in an increase in Bacteroidetes and a decrease in Firmicutes, which was consistent with the previous reports (Fig. S2B)^24^. Therefore, nicotine administration was demonstrated to alter the gut microbial composition specific to HFD feeding conditions.

### Changes in gut microbial metabolites induced by nicotine administration

**Figure 4:**
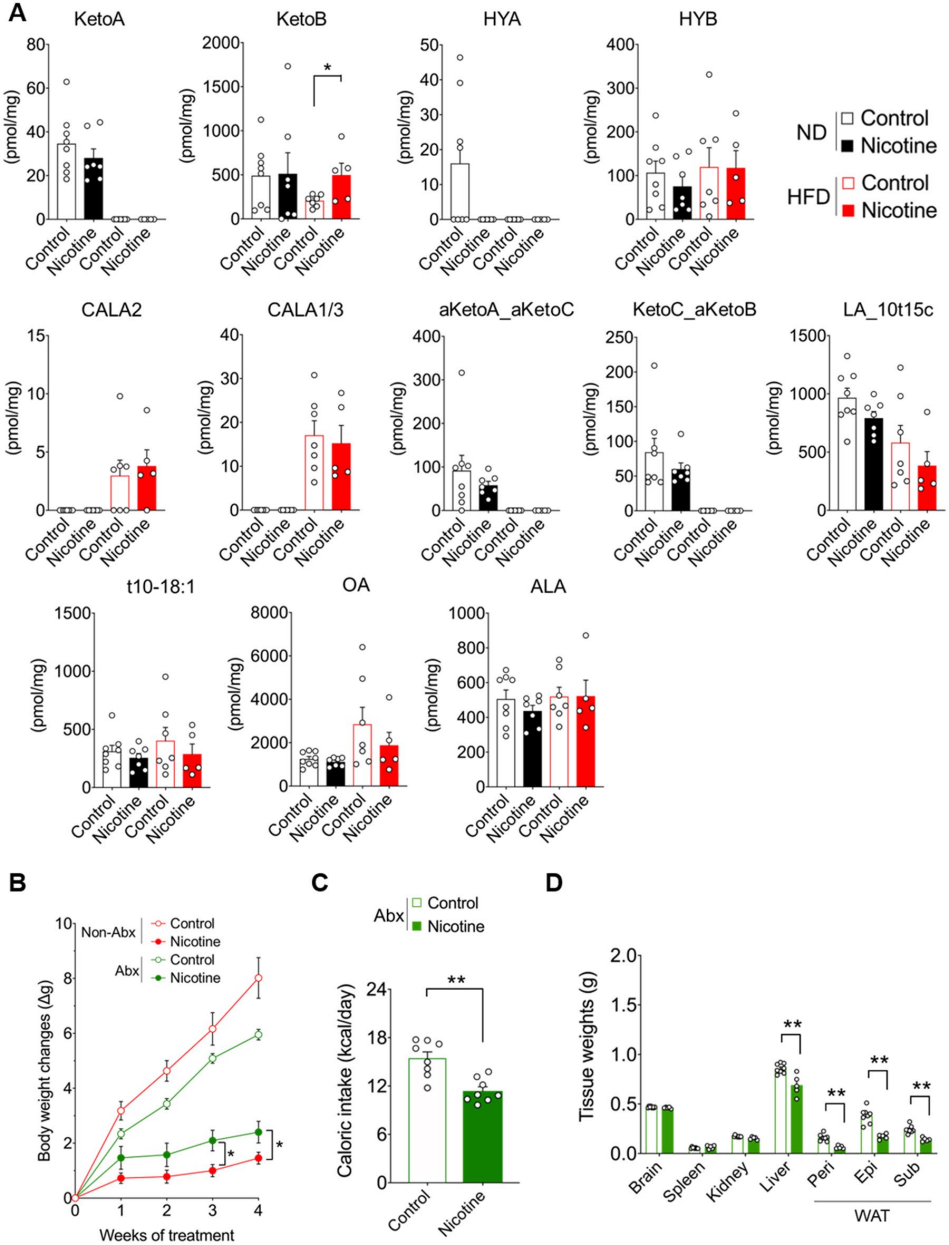
Changes in gut microbial metabolites in HFD-fed mice after the nicotine administration. (**A**) After the four-week period of intraperitoneal nicotine administration (1.5 mg/kg/day bodyweight), fatty acids-derived metabolites in the feces were determined (n = 5–8). For four weeks, antibiotics-treated C57BL/6J wild-type male mice were administered nicotine under HFD consumption. (**B**) Bodyweight gain during nicotine treatment (n = 8 per group), (**C**) daily caloric intake (n = 8 per group), (**D**) mass of the brain, spleen, kidney, liver, and WAT (n = 8 per group). All data are presented as the mean ± standard error of mean. *P < 0.01; *P < 0.05 (Student’s t-test: A; Two-way ANOVA followed by post hoc Bonferroni test: B; Mann–Whitney U test: C, D). KetoA, 10-oxo-*cis*-12-octadecenoic acid; KetoB, 10-oxo-octadecanoic acid; KetoC, 10-oxo-*trans*-11-octadecenoic acid; HYA, 10-hydroxy-*cis*-12-octadecenoic acid; HYB, 10-hydroxyoctadecanoic acid; OA, oleic acid; LA, linoleic acid; ALA, α-linolenic acid; ND, normal diet; HFD, high-fat diet; WAT, white adipose tissue; Peri, perirenal white adipose tissue; Epi, epididymal white adipose tissue; Sub, subcutaneous white adipose tissue; Abx, antibiotics.

Observational findings suggest that gut microbiota play an important role in maintaining human metabolic health^1,2^. Accumulating evidence suggests that gut microbial metabolites are a major source of molecular determinants that shape host–microbe interactions^3^. Significant increases in the abundance of *Lactobacillus* genera following nicotine administration were observed under HFD conditions alone. *Lactobacillus* is one of the predominant bacterial species found in the gastrointestinal tract. It can metabolize dietary linoleic and oleic acids, leading to the production of several hydroxylated or oxylated fatty acid metabolites, including 10-hydroxy-cis-12-octadecenoic (HYA), 10-hydroxy-octadecenoic (HYB), 10-oxo-cis-12-octadecenoic (KetoA), and 10-oxo-octadecenoic (KetoB) acids^4^. Therefore, we comprehensively analyzed and quantified the gut microbial metabolites following nicotine administration. Interestingly, among the metabolites, KetoB significantly increased in the HFD group alone after intraperitoneal nicotine administration (Fig. 4A), whereas HYB, which is also a modified fatty acid of saturated long-chain fatty acid (LCFA), showed no notable change. HYA and KetoA were not only undetectable in feces following HFD intake but HYA was also eliminated via intraperitoneal nicotine administration in the ND group. In contrast, there were no notable changes in the levels of fecal SCFAs, which are representative metabolites of gut bacteria in mice, independent of intraperitoneal nicotine administration, under both ND and HFD conditions (Fig. S3). Interestingly, the levels of SCFAs significantly increased in mice administered oral nicotine (Fig. S4), suggesting a possible link to the differences in the gut microbiota observed between intraperitoneal and oral nicotine administration (Fig. 3B and S2B). Finally, we examined the effects of nicotine administration on the metabolic characteristics under conditions that excluded the influence of gut bacteria and their metabolites. When a cocktail of four antibiotics (ampicillin, neomycin, metronidazole, and vancomycin) was administered ad libitum for four weeks to mice fed HFD, nicotine administration resulted in a significant reduction in caloric intake, and consequently, loss of body weight and mass of the liver and WAT. However, the degree of weight loss was substantially suppressed compared with that in the antibiotic-free group (Fig. 4B–D). These results suggest that gut microbial metabolites including KetoB, as a potential molecular entity, may play a role in the weight-reducing effects of nicotine administration under HFD feeding conditions.

## Discussion

Cigarette smoke is a complex mixture of chemicals, including nicotine, aldehydes, polycyclic aromatic hydrocarbons, nitrosamines, and heavy metals that is inhaled into the lungs as aerosol particles or absorbed through the skin and gastrointestinal tract^25^. Nicotine can accumulate in the upper digestive tract, which may have pathological implications as it has been linked to various smoking-related diseases^26^. Although nicotine plays a crucial role in regulating energy balance and metabolic homeostasis via diverse and simultaneous biological processes^13^, its impact on the gut microbiota under different dietary conditions remains unclear. Our findings mechanistically highlight the intensive cooperation among the gut microbiota, their metabolites, and host metabolic characteristics following nicotine treatment in a diet-dependent manner. We demonstrated that intraperitoneal nicotine administration has potential effects on weight regulation and metabolic phenotypes, independent of the effects attributable to reduced caloric intake. Furthermore, our results suggest that nicotine-induced body weight suppression was defined by the modulation of specific gut bacteria, including *Lactobacillus* spp., and provide further evidence indicating the involvement of KetoB, a linoleic acid-derived gut microbiota metabolite, during HFD intake alone.

Accumulating evidence indicates that nicotine decreases food intake and body weight via the hypothalamic melanocortin system and has uncovered key molecular and synaptic mechanisms involved in the appetite-suppressing effects of nicotine^14^. In the present study, the results of the pair-feeding model indicated that the mechanisms responsible for weight control induced by nicotine administration could not be solely attributed to a decrease in caloric intake. Furthermore, there was no precise correlation between caloric intake and body weight even under ad libitum conditions. Interestingly, although nicotine administration resulted in a comparable reduction in caloric intake in both ND and HFD groups, a notable reduction in body weight was observed specifically in the HFD group, suggesting the presence of potential diet-dependent factors contributing to weight loss induced by nicotine treatment. Recent studies have proposed that nicotine administration can alter specific gut microbiota populations, potentially playing a role in various physiological and pathophysiological processes^22,23^. Our findings indicate the importance of the gut microbiota and their dietary fatty acid-derived metabolites as key factors that potentially contribute to nicotine-induced body weight changes in a gut microbiota depletion model through antibiotic treatment in HFD-fed mice. Conversely, even in the antibiotic-treated mice, a significant reduction in caloric intake was observed following nicotine administration, indicating that the reduction in caloric intake caused by nicotine exposure cannot be solely attributed to gut bacteria. In addition, the control of energy metabolism by nicotine involving the endocrine system, including ghrelin, leptin, peptide YY (PYY), and glucagon-like peptide-1 (GLP-1), induced by nicotine exposure in peripheral tissues, has also been considered. However, evidences are less on the effect of nicotine on these food-regulating hormones and more rigorously controlled studies are needed in the future to resolve these outstanding issues^27^. Consequently, additional mechanisms underlying the weight-reducing effects of nicotine administration via the gut microbial community require further investigation.

In this study, we demonstrated that significant alterations in the gut microbiota following intraperitoneal nicotine administration were specifically observed under HFD alone, suggesting that elevated concentrations of LCFAs resulting from HFD intake indicate a potential impact on sensitivity to nicotine through modulation of the gut environment. Furthermore, the differences in the nicotine administration protocol may contribute not only to the alteration of gut microbial composition but also to the modulation of their metabolite profiles. Direct nicotine exposure in the intestinal tract via oral administration can alter the gut microbiota through physicochemical changes, including increased intestinal pH^28^. Conversely, nicotine intraperitoneal administration is considered to be absorbed into the systemic bloodstream and to indirectly affect the gut microbiota through nicotine receptors such as α7 nAChR, expressed in the central nervous system or enteric neural circuits^29^. Indeed, our results revealed that intraperitoneal administration of nicotine resulted in an increase in the abundance of Firmicutes, whereas oral administration led to a decrease in Firmicutes and a concomitant increase in Bacteroidetes, in accordance with a previous report^24^, consequently leading to an elevation in SCFA levels exclusively during oral administration. Further comprehensive investigations of the intricate regulatory mechanisms of the gut microbial community in response to nicotine exposure will provide insights into previously unknown mechanisms governing nicotine-induced host metabolic modulation.

KetoB, produced by *Lactobacillus* spp.^4^, has been suggested to exert anti-inflammatory effects^30^; however, previous studies have not investigated the impact of KetoB on host metabolic homeostasis. Our comprehensive quantitative analysis of LCFA-derived metabolites demonstrated, for the first time, the potential of KetoB as a regulator of body weight loss associated with nicotine administration. The relative abundance of *Lactobacillus* spp. populations exhibited a decrease in response to HFD exposure; however, an increase in their abundance was observed following nicotine administration, particularly under HFD conditions. These findings suggested a possible association between an augmented *Lactobacillus* population and enhanced KetoB production. However, to unveil the mechanism of the specific increase in KetoB, but not HYA, HYB, or KetoA, which are also metabolites of *Lactobacillus* spp., in response to nicotine administration in HFD-fed conditions, it is imperative to identify the gut microbial community, including the microbial populations and enzymes responsible for the metabolic pathway of KetoB, depending on the gut nutritional environment.

One of the primary mechanisms affecting the host metabolism of nicotine has been proposed to promote triglyceride lipolysis in adipose tissue and induce bodyweight loss, with concomitant increases in blood glucose levels in animal model of cigarette-extract exposure and tobacco smokers^15^. The mechanisms underlying these effects are considered to include the nicotine-mediated activation of AMP-activated protein kinases and a corresponding increase in serum free fatty acids via lipid anabolic processes, which contribute to increased insulin resistance^15,31^. Our results showed a significant increase in plasma NEFA levels following intraperitoneal nicotine administration in HFD-fed mice under ad libitum conditions, suggesting the potential role of nicotine in promoting lipolysis. However, blood glucose levels in mice treated with nicotine under HFD conditions were reduced. Possible reasons for these inconsistencies in the effects of nicotine on glucose homeostasis include the dosage and duration of nicotine administration, nicotine delivery systems, and specific responses in different tissues. Furthermore, the effect of nicotine administration on lipid absorption in the intestine should also be considered^32^. Our study revealed that the continuous intake of nicotine through drinking water, as opposed to acute intraperitoneal administration, nullified the significant reduction in blood glucose levels. These results indicate a potential link between the observed effects and differences in the gut microbial composition and metabolites following nicotine administration. Furthermore, the absence of significant changes in blood glucose levels and lipid metabolism parameters following intraperitoneal nicotine administration in the ND group, in contrast to the HFD group, suggests that the sensitivity of metabolic homeostasis to nicotine could be substantially modulated by the nutritional milieu within the body.

In this study, the weight management effects of nicotine administration were found to be diet-dependent, and the underlying mechanisms were attributed to the modulation of the gut microbiota, including the genus *Lactobacillus*, and the corresponding profile of a specific microbial metabolite, KetoB. Identifying the host receptor for KetoB produced by functional alterations in the gut microbiome in response to nicotine exposure and the subsequent regulation of metabolic homeostasis requires detailed follow-up investigations. Furthermore, characterizing a sensitive and comprehensive lipid metabolomic analysis of novel dietary lipid metabolites accompanying nicotine challenge may elucidate novel mechanisms of metabolic regulation based on the gut environment in a diet-dependent manner. Cigarette smoking affects the gut physiology and pathophysiology, including changes in the microbiome that affect systemic health. Our findings have likely highlighted the broad impact of the functional interplay between nutritional control and the gut environment in regulating host metabolism during smoking or smoking cessation. Further, these findings provide valuable insights into the promotion of significant interest in exploring therapeutic interventions targeting the microbiota for preventing and treating smoking-related diseases.

## Methods

### Animal study

Male C57BL/6J wild-type mice (Japan SLC, Shizuoka, Japan) were housed in a conventional animal room at 23.0 °C with a 12-h light–dark cycle. Before starting the experiments, the mice were acclimated to the laboratory conditions and fed the CLEA Rodent Diet (CE-2, CLEA Japan, Tokyo, Japan). At seven weeks of age, the male mice with comparable average bodyweight were divided into two groups: the ND group and the HFD group (D12492, 60% kcal fat; Research Diets, Inc., NJ, USA) and administered twice daily injections of nicotine (0.75 mg/kg bodyweight, intraperitoneal administration) or saline for four weeks^15^. After the intervention, fecal levels of SCFAs and long-chain fatty acid metabolites were determined. For the pair-feeding experiments, male mice were raised from 7 to 11 weeks of age. Each cage housed one mouse with a similar average weight, and their respective food intakes were measured. Control mice were pair-fed to match the food intake of nicotine-treated mice. Under these conditions, the control mice were fed the total amount of food consumed by the nicotine-administered mice on the previous day, delivered every 12 h the following day. For oral nicotine delivery model experiments, 7-week-old male mice were administered a nicotine solution (200 μg/mL in 2% saccharin vehicle) in drinking water, as reported previously^33^, during the four-week period of HFD exposure. For the antibiotic treatment, HFD-fed mice were treated with ampicillin (0.4 mg/mL, FUJIFILM Wako, Osaka, Japan), neomycin (0.4 mg/mL, Sigma-Aldrich, St. Louis, MO, USA), metronidazole (0.4 mg/mL, FUJIFILM Wako), and vancomycin (0.2 mg/mL, FUJIFILM Wako) in drinking water for four weeks. During the experiments, bodyweights were measured once a week. In all experimental animal models, the mice were dissected after 5 h of fasting. Blood samples were obtained from the inferior vena cava using heparin-treated tubes. The tubes were immediately centrifuged at 7000*g* for 5 min at 4 °C to separate the plasma. After nicotine administration, brain, spleen, kidneys, liver, WAT, and gastrointestinal tract were harvested, and their weights were compared.

### Plasma biochemical analyses

Blood glucose concentrations were assessed using a One Touch Ultra device from LifeScan (Milpitas, CA, USA). The plasma levels of total cholesterol, NEFAs, and TGs were measured using commercially available kits (LabAssay Cholesterol for total cholesterol, LabAssay NEFA for NEFAs, and LabAssay Triglyceride for TGs; FUJIFILM Wako), following a previously described protocol^34^.

### Quantification of gut microbial metabolites by liquid chromatograph-mass spectrometry (LC–MS/MS)

The gut microbial metabolites of LCFAs used in this study were synthesized according to previously published methods with a minimum purity of 98%^5^. To measure individual fatty acid-derived metabolites, approximately 50 mg of fecal content was homogenized in 1 mL of methanol, and an internal control (C19:0) was added to the samples. Next, 2 mL of chloroform and 0.75 mL of 0.5 M potassium chloride were added to extract the lipids. The lipid layers were collected, dried, and reconstituted in a mixture of chloroform and methanol (1:3, v/v) for LC–MS/MS analysis. Fatty acid-derived metabolites were analyzed using an Acquity UPLC system coupled with a Waters Xevo TQD mass spectrometry (Waters, Tokyo, Japan) equipped with ACQUITY UPLC BEH C18 column (2.1 × 150 mm, 1.7 μm; Waters). Multiple-reaction-monitoring parameters were optimized for each target compound using standards. Quantitation was conducted using calibration curves specific to each compound, and the recoveries were monitored using deuterated internal standards.

### Measurement of SCFA by gas chromatography-mass spectrometry (GC/MS)

Fecal SCFAs were determined using a modified procedure, as described previously^35^. The ether layers containing SCFAs were combined and subjected to GC–MS analysis using a GCMS-QP2010 Ultra instrument (Shimadzu, Kyoto, Japan). The concentration of SCFAs in each sample was quantified by employing an external standard calibration across a defined concentration range.

### Analysis of gut microbiota by 16S rRNA gene sequencing

Fecal DNA was extracted from frozen samples using the FastDNA® SPIN Kit for Feces (MP Biomedicals, Santa Ana, CA, USA). The V4 region of the 16S rRNA gene was amplified using dual-indexed primers. Sequencing of the amplicons was carried out on an Illumina MiSeq instrument with a MiSeq Reagent Kit V3 (Illumina, San Diego, CA, USA), following established protocols^36^. Read processing and quality filtering were performed using Quantitative Insights into Microbial Ecology (QIIME) version 1.9.1. and aligned with the SILVA database (http://www.arb-silva.de) at the Unclassified level. Further analysis at each taxonomic level involved data normalization, and identification of differentially abundant taxa was based on the criteria of a false discovery rate (FDR)-adjusted p-value < 0.05, using the Benjamini–Hochberg procedure. PCA plots and heatmap-based clustering analysis were conducted using the prcomp and heatmap functions, respectively, in the R package. These analyses aimed to detect and visualize clustering patterns at each taxonomic level.

### Statistical analysis

All data are presented as mean ± standard error of the mean. GraphPad Prism version 8.1.0 (GraphPad Software Inc., La Jolla, CA, USA) was used for the statistical analyses. Data normality was assessed using the Shapiro–Wilk test. For the statistical comparisons, the Student’s t-test (two-tailed) or the Mann–Whitney U test (two-tailed), or one-way analysis of variance (ANOVA) followed by Tukey’s test or the Kruskal–Wallis test followed by Dunn’s post-hoc test, or two-way ANOVA with the Bonferroni test was applied as appropriate, depending on data normality. Statistical significance was set at *P* < 0.05. Permutational multivariate analysis of variance tests were performed to assess the similarity of microbiomes. The FDR q-values in 16S rDNA sequencing were estimated using the Benjamini–Hochberg procedure.

### Ethical approval

All mouse-associated procedures were conducted in accordance with the approved protocols of the Kyoto University Animal Experimentation Committee (Lif-K22015) and the Tokyo University of Agriculture and Technology (Permit no. 28-87) for ethical treatment of animals. All methods were approved by the Kyoto University Animal Experimentation Committee (Lif-K22015) and the Tokyo University of Agriculture and Technology (Permit no. 28-87). Mice were euthanized under deep anesthesia using isoflurane, and every effort was made to minimize any potential discomfort or suffering experienced by the animals. All experiments were performed in accordance with ARRIVE guidelines (https://arriveguidelines.org).

### Supplementary Information

Supplementary Figures.

## Data Availability

The data that support the findings of this study are available from the corresponding author on reasonable request. The 16S rRNA gene sequence data have been deposited in the DNA Data Bank of Japan (DDBJ) under the accession no. DRA016737, and the Dryad repository (10.5061/dryad.rxwdbrvfh).
