# Supplementary material for: Gut microbial metabolites reveal diet-dependent metabolic changes induced by nicotine administration
Source: Sci Rep. 2024 Jan 11;14:1056. doi: 10.1038/s41598-024-51528-3 (PMC10784489; doi:10.1038/s41598-024-51528-3)
Supplement: Supplementary file 1 — Supplementary Figures. [file 41598_2024_51528_MOESM1_ESM.pdf]

SUPPLEMENTARY INFORMATION

Gut microbial metabolites reveal diet-dependent metabolic changes induced by nicotine administration

Ryuji Ohue-Kitano, Yukika Banno, Yuki Masujima, and Ikuo Kimura

Corresponding author:
Ryuji Ohue-Kitano
Ikuo Kimura

This file includes:
Figures S1–S4

Supplemental Figure 1

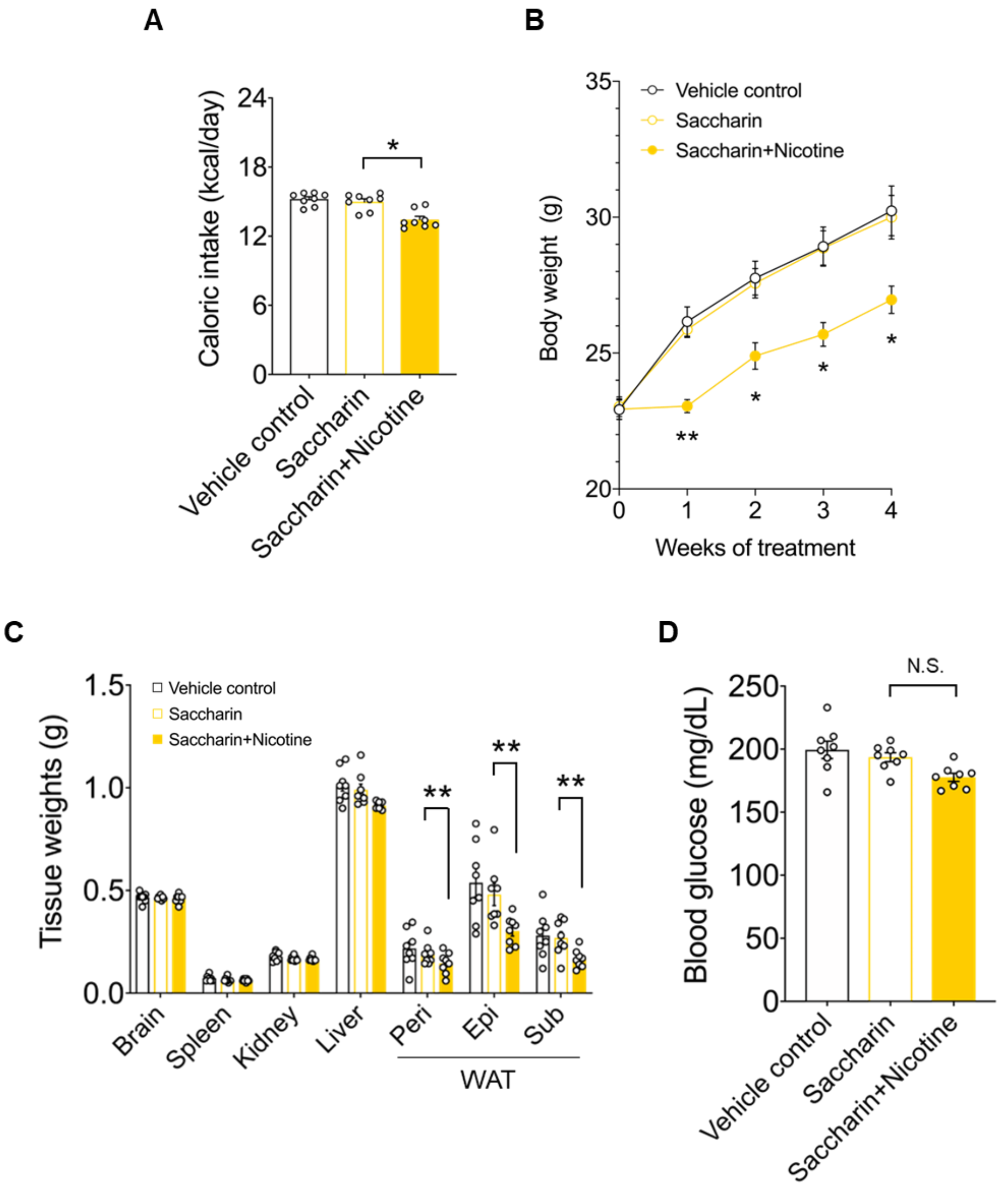

Supplemental Figure 1. The effect of oral nicotine administration on host metabolism. 7-week-old C57BL/6J wild-type male mice were received a nicotine in drinking water (200 μg/mL in 2% saccharin vehicle) during the 4-week period of HFD exposure. (**A**) Daily caloric intake (n = 8 per group), (**B**) bodyweight gain (n = 8 per group), (**C**) mass of the brain, spleen, kidney, liver, and WAT (n = 8 per group), and (**D**) blood glucose levels. All data are presented as the mean ± standard error of mean. **P < 0.01; *P < 0.05 (Kruskal–Wallis test followed by post hoc Dunn's test: A; Two-way ANOVA followed by post hoc Bonferroni test: B; One-way ANOVA followed by post hoc Tukey's test: C, D). WAT, white adipose tissue; Peri, perirenal white adipose tissue; Epi, epididymal white adipose tissue; Sub, subcutaneous white adipose tissue; N.S., not significant.

Supplemental Figure 2

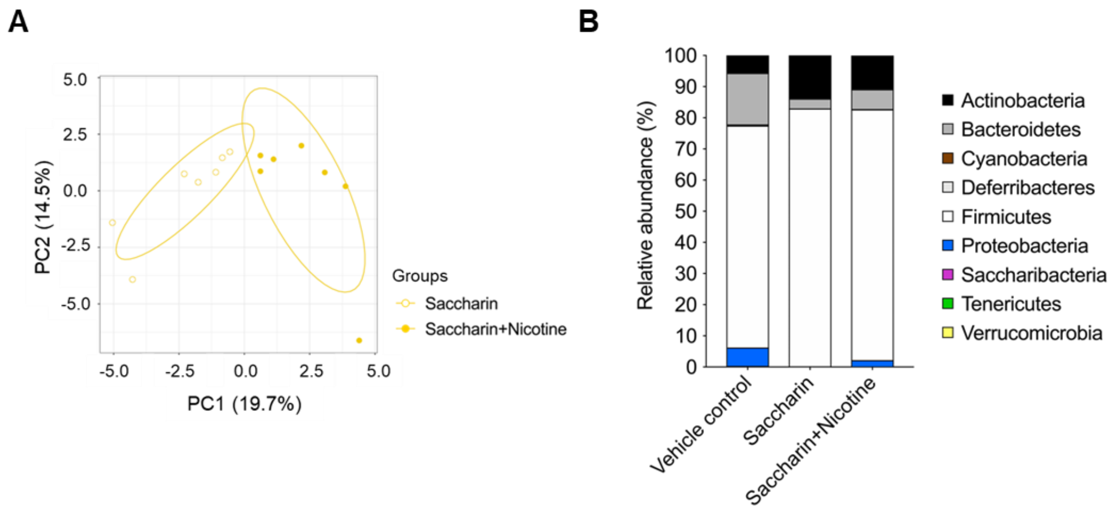

Supplemental Figure 2. The effect of oral nicotine administration on gut microbiota composition. 7-week-old C57BL/6J wild-type male mice were received a nicotine in drinking water (200 μg/mL in 2% saccharin vehicle) during the 4-week period of HFD exposure. (**A**) Principal coordinate analysis of the fecal microbiota and (**B**) relative abundance of the phylum level (n = 7 per group). Permutational multivariate analysis of variance tests were used to analyze the similarity of microbiomes. All data are presented as the mean ± standard error of mean.

Supplemental Figure 3

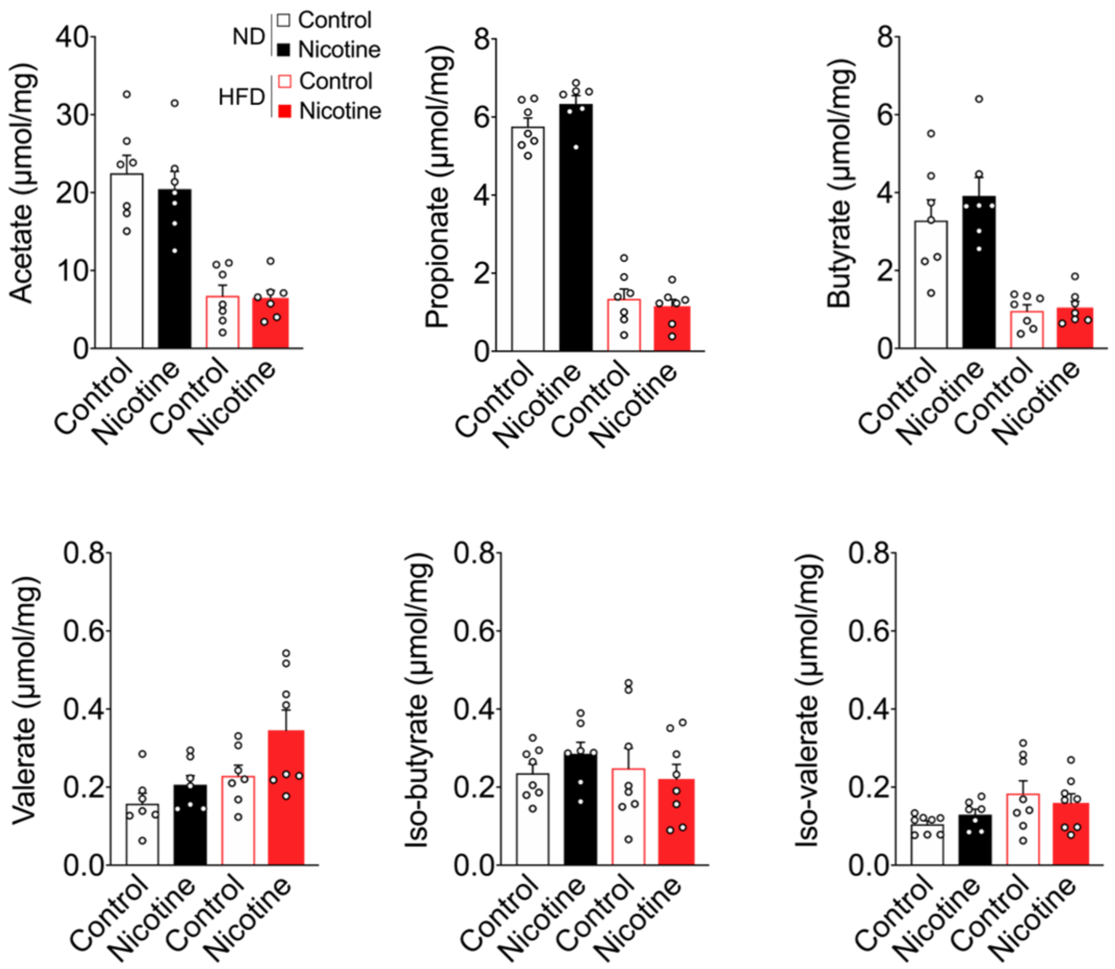

Supplemental Figure 3. Fecal SCFA levels in mice fed ND or HFD during intraperitoneal nicotine administration. After the 4-weeks period of intraperitoneal nicotine administration (1.5 mg/kg/day bodyweight), SCFA levels in the feces were determined (n = 7–8). All data are presented as the mean ± standard error of mean. ND, normal diet; HFD, high-fat diet; SCFA, short-chain fatty acid.

Supplemental Figure 4

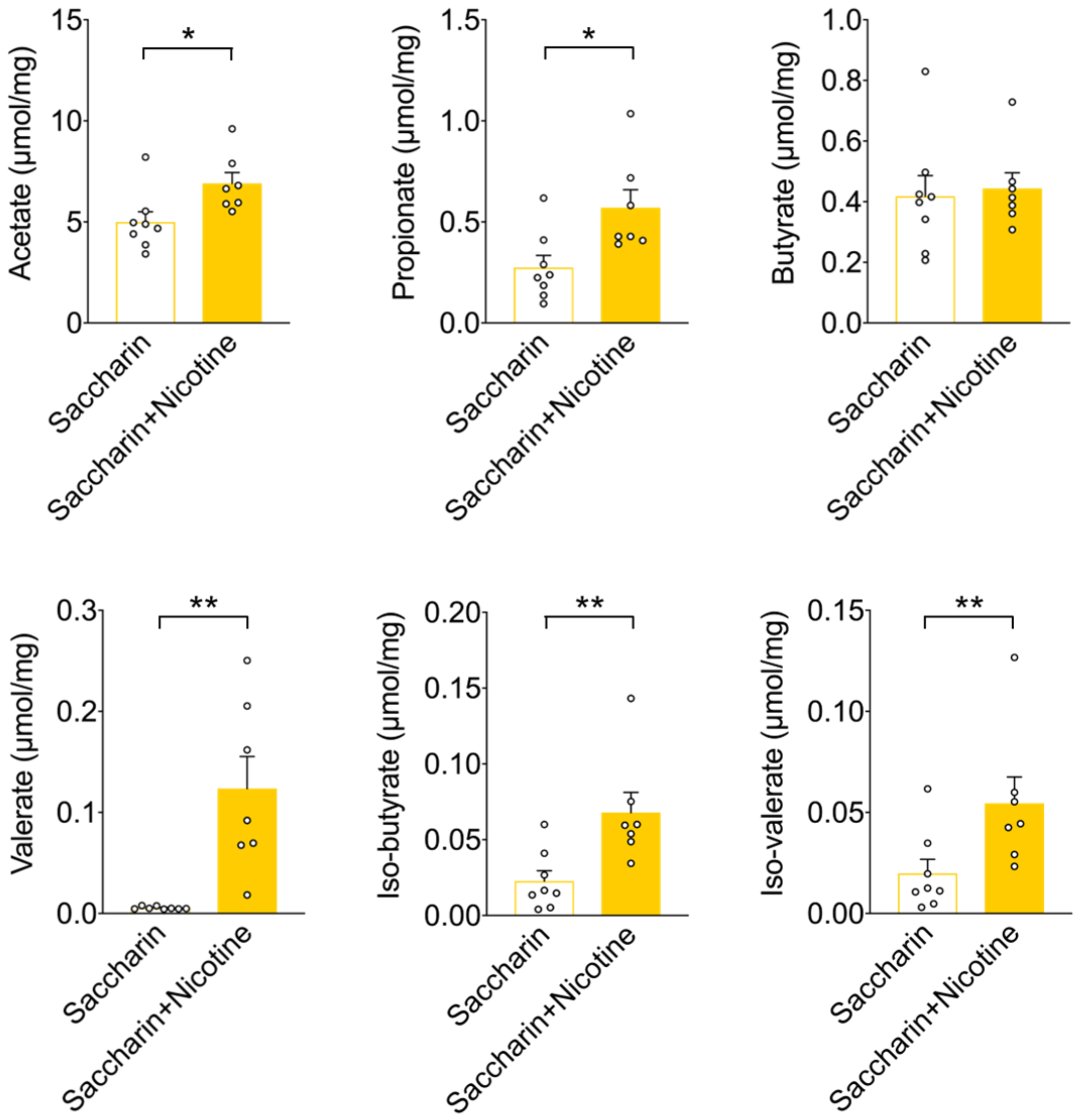

Supplemental Figure 4. Fecal SCFA levels in mice fed HFD during orally nicotine exposure. 7-week-old C57BL/6J wild-type male mice were received a nicotine in drinking water (200 µg/mL in 2% saccharin vehicle) during the 4-week period of HFD exposure. After the nicotine intervention, SCFA levels in the feces were determined (n = 7–8). All data are presented as the mean ± standard error of mean. $^{**}P < 0.01$; $^{*}P < 0.05$ (Mann-Whitney U test). HFD, high-fat diet; SCFA, short-chain fatty acid.
